# Identifying Phenogroups in patients with subclinical diastolic dysfunction using unsupervised statistical learning

**DOI:** 10.1186/s12872-020-01620-z

**Published:** 2020-08-14

**Authors:** Yvonne E. Kaptein, Ilya Karagodin, Hongquan Zuo, Yu Lu, Jun Zhang, John S. Kaptein, Jennifer L. Strande

**Affiliations:** 1 Aurora Cardiovascular Services, Aurora St Luke’s Medical Center, Milwaukee, WI USA; 2 Department of Medicine, Medical College of Wisconsin, Milwaukee, USA; 3 Department of Medicine, University of Chicago, Chicago, USA; 4 Electrical Engineering and Computer Science, University of Wisconsin-Milwaukee, Milwaukee, USA; 5 University of Southern California, Los Angeles, USA; 6 Cardiovascular Center, Medical College of Wisconsin, Milwaukee, USA

**Keywords:** Diastolic dysfunction, Heart failure with preserved ejection fraction, Unsupervised machine learning, Hierarchical clustering, Clinical studies, Risk factors, Heart failure, Echocardiography

## Abstract

**Background:**

Subclinical diastolic dysfunction is a precursor for developing heart failure with preserved ejection fraction (HFpEF); yet not all patients progress to HFpEF. Our objective was to evaluate clinical and echocardiographic variables to identify patients who develop HFpEF.

**Methods:**

Clinical, laboratory, and echocardiographic data were retrospectively collected for 81 patients without HF and 81 matched patients with HFpEF at the time of first documentation of subclinical diastolic dysfunction. Density-based clustering or hierarchical clustering to group patients was based on 65 total variables including 19 categorical and 46 numerical variables. Logistic regression analysis was conducted on the entire study population as well as each individual cluster to identify independent predictors of HFpEF.

**Results:**

Unsupervised clustering identified 3 subgroups which differed in gender composition, severity of cardiac hypertrophy and aortic stenosis, NT-proBNP, percentage of patients who progressed to HFpEF, and timing of disease progression from diastolic dysfunction to HFpEF to death. Clusters that had higher percentages of women had progressively milder cardiac hypertrophy, less severe aortic stenosis, lower NT-proBNP, were diagnosed at an older age with HFpEF, and survived to an older age. Independent predictors of HFpEF for the entire cohort included diabetes, chronic kidney disease, atrial fibrillation, and diuretic use, with additional predictive variables found for each cluster.

**Conclusions:**

Cluster analysis can identify phenotypically distinct subgroups of patients with diastolic dysfunction. Clusters differ in HFpEF and mortality outcome. In addition, the variables that correlate with and predict HFpEF outcome differ among clusters.

## Background

Left ventricular (LV) diastolic dysfunction is characterized by alterations in LV diastolic filling, and is a strong predictor of cardiovascular events including heart failure and its subtype heart failure with preserved ejection fraction (HFpEF) [1]. The prevalence of HFpEF has increased over the past decades but the death rate has not changed substantially [1]. Several risk factors including age, obesity, hypertension, diabetes mellitus, chronic kidney disease, and coronary artery disease are implicated in the development of diastolic dysfunction as well as HFpEF [1–3]. Importantly, asymptomatic diastolic dysfunction precedes the development of HFpEF; yet, not all patients with diastolic dysfunction will progress to clinical or symptomatic HFpEF, possibly due to the phenotypic heterogeneity of this population. HFpEF is also a heterogeneous disease with similar predisposing risk factors and associated comorbidities [2]. HFpEF suffers from lack of any standardized therapies that effectively reduce mortality [2, 4–6] and therefore, the prevention of HFpEF remains a goal. This highlights the importance of understanding the risk factors associated with progression from diastolic dysfunction to HFpEF, as a path towards improving prognostication of the disease, personalizing therapy, and ultimately improving clinical outcomes, namely progression to clinical HFpEF or overall mortality.

Machine learning can be used to apply computer analysis to large data sets to identify patterns and trends. The goal of unsupervised machine learning, or cluster analysis is to learn the relationships between variables and uncover a hidden structure in the data set. It relies on clustering and dimensionality reduction. Due to the complexity of the data and heterogeneity of patients in medicine, intuitively identifying groups with similar phenotypes can be difficult and therefore the ability to identify these groups using machine learning methods may allow for more targeted diagnostics, therapeutic strategies and prognostication. For example, unsupervised machine learning has been previously used in research to divide large heterogeneous populations of patients into smaller unique phenogroups, including patients with HFpEF [7, 8], patients with primary hypertension (HTN) without heart failure [9], and mixed patient groups with HFrEF and HFpEF combined [10, 11]. In general, machine learning is a process that uses statistical algorithms to allow computers to learn relationships between objects or in these examples, patients, based on degree of similarities or differences among any number of categorical or quantitative variables, allowing the learning algorithm to find structure or hidden patterns in uncategorized data [12]. To our knowledge, unsupervised learning has not previously been described in the literature to analyze patients with asymptomatic diastolic dysfunction.

Here we describe the use of unsupervised machine learning and hierarchical clustering to identify subgroups of patients with asymptomatic diastolic dysfunction who have similar phenotypes. The intent was to cluster patients with similar physical and clinical characteristics regardless of whether they progressed to HFpEF or remained asymptomatic, following which the characteristics of the clusters were to be examined. We identified the features specific to each cluster and determined those which are independently predictive of developing HFpEF. We also evaluated the differences in phenotypes among clusters, along with the differences between patients within each cluster who were known to progress or not progress to clinical HFpEF. Lastly, we used survival curve analysis to identify differences in disease progression and mortality outcomes among clusters.

## Methods

### Study design and patient data collection

This was an Institutional Review Board approved study conducted at the Medical College of Wisconsin and at Froedtert Memorial Lutheran Hospital in Milwaukee, WI. No informed consent was required. This is a matched retrospective case-control study; subject screening and selection was described in detail elsewhere [13]. In brief, patients were first identified by screening transthoracic echocardiograms (TTEs) obtained between 7/1/2003 and 7/1/2013 reporting diastolic dysfunction and preserved ejection fraction (EF > 50%). Patients were excluded if they had systolic dysfunction (EF < 50%), valve abnormalities including severe aortic stenosis, severe mitral regurgitation, annuloplasty, and/or bioprosthetic valves, a heart transplant or non-diagnostic echocardiograms.

The remaining patients were further sub-divided into two groups: (1) those who had clinical heart failure during the study period, and (2) those who remained in asymptomatic diastolic dysfunction. Patients with clinical heart failure were identified if their electronic health record contained an ICD-9 diagnosis of congestive heart failure along with clinical documentation of at least one of the following signs or symptoms of heart failure: shortness of breath, orthopnea, paroxysmal nocturnal dyspnea, weight gain, or lower extremity edema. Patients in Group 1 diagnosed with HFpEF were optimally matched for age, gender, race, and body surface area (BSA) with Group 2 patients who remained in asymptomatic diastolic dysfunction. This ultimately yielded 77 matched pairs which were included in our study. Later, an additional screen added 8 patients with diastolic dysfunction (half of which were known to develop HFpEF) to the study population. It was impractical to extract data for all patients initially screened for diastolic dysfunction.

Our study population therefore contained 162 patients, all of whom had TTE evidence of diastolic dysfunction and normal EF, but only half of whom progressed to develop clinical HFpEF. TTE reports were retrospectively screened to find the earliest documentation of diastolic dysfunction prior to any diagnosis of HFpEF. Numerical data from this earliest TTE were utilized, including systolic and diastolic blood pressure (SBP/DBP), and several measured and calculated echocardiographic parameters including degree of diastolic dysfunction. In addition, numerous other qualitative and quantitative demographic and clinical data points were collected for each patient for use in the unsupervised learning cluster analysis. Supplemental Table 1 contains the list of variables used for clustering analysis and all authors have full access to this data and take responsibility for its integrity and the data analysis.

While gender, race, body surface area, and age of diastolic dysfunction may play a role in progression to HFpEF, these are factors that cannot be modified. This study was intended to analyze variable factors that could potentially be treated or controlled, and whether these factors might differ for phenotypically different types of patients. Matching of patients among the two cohorts was done in order to discern the effect, if any, of variable factors.

For subsequent analysis of mortality and survival outcomes in all 162 patients, the cutoff date of 5/5/2018 was used to determine which patients were alive or deceased at the time of data analysis. Patients were considered “deceased” if it was indicated in the chart that the patient had died, if there was a date of death listed, or if the most recent notes in the electronic health record indicated a date of confirmed death. Otherwise, the most recent notes were scanned for mention of a face-to-face encounter with the patient, or a telephone conversation with the patient or family member discussing further plans of care, and this date was used as a “last known alive” date for survival analysis.

### Unsupervised hierarchical clustering of patients

Unsupervised machine learning was used to group patients with asymptomatic diastolic dysfunction into clusters with similar physical and clinical characteristics without regard to either progression to HFpEF or mortality outcomes. One patient with HFpEF was excluded from clustering analysis due to significant outliers (left ventricular outflow tract (LVOT) velocity max, LVOT velocity mean, LVOT max gradient, and LVOT mean gradient) which were 8 standard deviations larger than the mean. We used either density-based clustering or hierarchical clustering to group patients based on 65 total variables including 19 categorical and 46 numerical variables (Supplemental Table 1). Heart failure and survival data were excluded from the clustering analysis. Each categorical variable was converted into a numerical one through one-hot encoding. If the categorical data contained multiple categories (i.e. Chronic kidney disease stage 1–5 and degree of diastolic dysfunction: mild, moderate or severe) then these were treated as dichotomous data (yes or no) and then converted into numerical data using one-hot encoding. Missing data points were estimated and imputed using a singular value decomposition technique for data analysis [14]. The squared Euclidean distances between each pair of patients were calculated and put into a distance matrix, which served as the input into the hierarchical clustering algorithm. Two-, three-, four-, and five-cluster determinations were achieved with each patient being assigned to one of the clusters. To determine the optimal number of clusters or phenogroups we performed a chi-square analysis to look for significant differences between clusters in each row. The resultant clusters were then statistically analyzed for any differences in their variable composition or phenotype, and heart failure and mortality outcomes. The purity of the clustering distribution was calculated by determining the percentage of total patients whose HFpEF outcome agreed with the majority of patients in the cluster to which the patient was assigned. Purity was calculated as:

$$ \mathrm{purity}=\frac{1}{N}\sum \limits_{k=1}^K{n}_k $$

where *N* is the total number of patients, *k* represents the *k*th cluster, *n*_*k*_ is the number patients in the *k*th cluster that has the majority status in terms of presence or absence of heart failure [15].

### Statistical analysis of data

Before clustering, logistic regression analysis was conducted on the entire population to distinguish which variables were predictors of HFpEF outcome. Univariate logistic regression was used in all cases as a first determination of which variables were significant predictors of progression. Those variables which were significant predictors on their own were then progressively added to the prediction. Whenever there was collinearity, the variable with the most significance was retained and the one with the lesser significance was discarded. The variables remaining after this process were all independent of each other and were all statistically significant predictors. With only a limited size dataset, only a limited number of variables could be expected to be found significant.

Two-fold cross validation of the predictive modeling was performed. Each cohort was divided into two halves by assigning alternating members of the cohort to either a discovery group or a validation group. Since sample size was small, variables for the cross validation study were assumed to be those found for the entire cohort. Coefficients for the logistic regressions were recalculated for the discovery group. Predictions were then made as to whether each patient of the discovery cohort and of the validation cohort was predicted to remain asymptomatic or progress to HFpEF. Predictions for the validation group were compared to the predictions for the discovery group. The process was repeated using the second half as the discovery group and the first half as the validation group.

Once clusters were identified, we examined the phenotypic variables within each group. Continuous data were presented as mean +/− 95% confidence interval. Categorical variables were presented as a count or percentage. We compared differences between groups using chi-square test for categorical variables and analysis of variance (ANOVA) for continuous variables. Likewise, the percentage of patients who remained asymptomatic vs. those who developed HFpEF were also compared among groups using chi-square test. If ANOVA showed statistically significant differences in one variable among several groups, then the Newman-Keuls multiple comparisons test or Duncan’s multiple comparisons test was used when appropriate to find which cluster contained the variable that was significantly different.

Each cluster contained patients that remained asymptomatic or developed HFpEF. We compared differences between these two subgroups within each cluster by using chi-square test for categorical variables and ANOVA for continuous variables. Logistic regression was used to analyze the data for independent predictors of HFpEF for each cluster.

Key prognostic factors such as age of diastolic dysfunction diagnosis, time interval between diagnosis of diastolic dysfunction and HFpEF, age of HFpEF diagnosis, time interval between diagnosis of HFpEF until death due to all causes, and age at death were analyzed by Kaplan-Meier survival curves to identify differences in disease progression within and among clusters. For survival analysis, actual date of an event or last known date without the event occurring were recorded (right censored data) using the cutoff date of 5/5/2018.

Statistics were performed using Microsoft Office Excel 2010 or Epistat version 5.3 (Epistat Services, Richardson TX) and graphs were generated using SigmaPlot version 13, Systat Software Inc., San Jose, CA.

## Results

### Characteristics of the subclinical diastolic dysfunction study population

We retrospectively enrolled 162 patients for our phenogrouping analysis. The patients were matched given that half the patients were known to progress to HFpEF. The goal was to identify phenogroups of patients with subclinical diastolic dysfunction and to determine distinguishing characteristics that are predictive of progression to HFpEF.

Demographic characteristics of the study cohort are shown in Table 1, which compares patients who remained in asymptomatic diastolic dysfunction to those patients who progressed to HFpEF within the time period of the study. Overall, patients were diagnosed with subclinical diastolic dysfunction at age 70 ± 10 years and of these, those patients who were known to develop HFpEF were diagnosed at age 74 ± 10 years. The cohort contained 67.9% female, and 77.8% white patients. Patients who progressed to HFpEF were more likely to have a history of diabetes mellitus (DM), chronic kidney disease (CKD), and atrial fibrillation (Afib), as well as the use of digoxin and diuretics. In contrast, the use of aldosterone antagonists was more prevalent in the patient cohort who remained in asymptomatic diastolic dysfunction. The N-terminal pro hormone BNP (NT-proBNP) levels and degree of diastolic dysfunction severity also differed between the cohort whoremained in asymptomatic diastolic dysfunction and the cohort who progressed to HFpEF; the cohort who progressed to HFpEF had a higher average NT-proBNP but contained more patients with mild diastolic dysfunction.

**Table 1:** Population Demographics

Patient Characteristics	Subclinical	Outcomes		
	Diastolic Dysfunction Total Cohort (*n* = 162)	Subclinical Group 1 (*n* = 81)	HFpEF Group 2 (*n* = 81)	*p*-value* Group 1 vs. Group 2
Age at diagnosis of Diastolic Dysfunction	69.6 ± 10.1	68.8 ± 9.9	70.5 ± 10.3	0.289
Gender (% female)	67.9%	67.9%	67.9%	1.000
Race (% White, remainder Black)	77.8%	77.8%	77.8%	1.000
Weight (lb)	194.9 ± 59.3	193.5 ± 53.5	196.4 ± 64.9	0.757
Height (in)	65.6 ± 4.2	65.6 ± 4.1	65.6 ± 4.4	0.985
Body surface area (m^2^)	1.95 ± 0.28	1.95 ± 0.27	1.95 ± 0.30	0.987
Interval until diagnosis of HFpEF (years)	N/A	N/A	5.4 ± 2.8	
Age at diagnosis of HFpEF	N/A	N/A	74.0 ± 10.5	
Age at death OR last known alive	76.8 ± 10.3	76.0 ± 9.9	77.6 ± 10.7	0.319
Mortality, n (%)	66 (40.1)	26 (32.1)	40 (49.4)	0.038
NT-Pro B-type natriuretic peptide (pg/mL) (n)	8178 ± 14,346 (72)	2148 ± 3026 (21)	10,661 ± 16,341 (51)	0.021
Diastolic Dysfunction Severity (n)	(*n* = 162)	(*n* = 81)	(*n* = 81)	
Mild: E/A < 1, average e’ ≤ 9 cm/s	28 (17.3%)	7 (8.6%) ↓	21 (25.9%) ↑	0.011
Moderate: E/A ≥ 1, average e’ ≤ 9 cm/s	125 (77.2%)	68 (84.0%)	57 (70.4%)	
Severe: E/A ≥ 2, average e’ ≤ 9 cm/s	9 (5.6%)	6 (7.4%) ↑	3 (3.7%) ↓	
Degree of cardiac hypertrophy (n)	(*n* = 152)	(*n* = 77)	(*n* = 75)	
None	90 (59.2%)	51 (66.2%) ↑	39 (52.0%) ↓	0.053
Mild	26 (17.1%)	15 (19.5%)	11 (14.7%)	
Moderate	14 (9.2%)	4 (5.2%) ↓	10 (13.3%) ↑	
Severe	22 (14.5%)	7 (9.1%) ↓	15 (20.0%) ↑	
Chronic Kidney Disease Stage (n)	(*n* = 147)	(*n* = 70)	(*n* = 77)	
Glomerular filtration rate (GFR)				0.100
Stage 1–2 GFR > 60 mL/min/1.73m^2^)	63 (42.9%)	34 (48.6%)	29 (37.7%)	
Stage 3a (GFR 45–59 mL/min/1.73m^2^)	32 (21.8%)	18 (25.7%)	14 (18.2%)	
Stage 3b (GFR 30–45 mL/min/1.73m^2^)	26 (17.7%)	8 (11.4%)	18 (23.4%)	
Stage 4 (GFR 15–30 mL/min/1.73m^2^)	11 (7.5%)	6 (8.6%)	5 (6.5%)	
Stage 5a (GFR < 15, mL/min/1.73m^2^)	15 (10.2%)	4 (5.7%)	11 (14.3%)	
History of co-morbidities, (n)	*n* = 151 to 157	n = 75 to 76	*n* = 76 to 81	
Hypertension	81.5%	76.3%	86.4%	0.154
Diabetes	42.0%	27.6%	55.6%	< 0.001
Chronic kidney disease	44.2%	25%	62.5%	< 0.001
Alcohol use	53.6%	58.7%	48.7%	0.286
Tobacco use	56.1%	57.9%	54.4%	0.785
Coronary artery disease	51.3%	42.7%	59.3%	0.056
Cerebral vertebral accident/transient ischemic attack	18.1%	13.3%	22.5%	0.203
Atrial fibrillation	29.3%	19.7%	38.3%	0.018
Medication Use by Class (n)	*n* = 154 to 155	*n* = 73 to 74	*n* = 81	
Beta blockers	68.2%	63.0%	72.8%	0.257
Calcium channel blockers	27.3%	28.8%	25.9%	0.831
ACE inhibitors	23.4%	17.8%	28.4%	0.174
Angiotensin blockers	18.2%	15.1%	21.0%	0.458
Digoxin	5.8%	0.0%	11.1%	0.009
Diuretics	52.6%	35.6%	67.9%	< 0.001
Aldosterone antagonist	11.7%	19.2%	4.9%	0.013

n = total number of patients in each cohort or n = number of patients with available data

*categorical values are presented as counts and percentages; continuous variables are presented as mean ± 95% confidence interval

↑ indicates higher than expected by chance

↓ indicates lower than expected by chance

### Risk predictors of developing HFpEF in patients with subclinical diastolic dysfunction

For the entire 162 patient cohort, four categorical variables (history of DM, CKD, AFib, and diuretic use) were found to be independent and statistically significant (*p* < 0.05) positive predictors of development of clinical HFpEF while adjusting for other factors. Using logistic regression, the probability (***P***) of a patient in our population developing heart failure, based on the presence or absence of these four factors while adjusting for other factors is:

$$ \boldsymbol{P}=\frac{\mathbf{1}}{\mathbf{1}+{\boldsymbol{e}}^{\left(\mathbf{1.94}-{\left(\mathbf{0}\ \boldsymbol{or}\ \mathbf{1.14}\right)}_{\boldsymbol{DM}}-{\left(\mathbf{0}\ \boldsymbol{or}\ \mathbf{1.37}\right)}_{\boldsymbol{CKD}}-{\left(\mathbf{0}\ \boldsymbol{or}\ \mathbf{1.08}\right)}_{\boldsymbol{AFib}}-{\left(\mathbf{0}\ \boldsymbol{or}\ \mathbf{1.39}\right)}_{\boldsymbol{Diuretic}}\right)}} $$

Coefficients for category variables are interpreted as 0 if the patient has a negative history, or the indicated value in the above equation if the patient has a positive history. We have complete data for these four variables in 151 patients and these patients have an incidence of HFpEF of 53.0%. Therefore, a prediction probability (***P***) greater than 53.0% predicts the development of HFpEF, and a ***P*** less than this predicts the patients will remain asymptomatic. The sensitivity and specificity for this prediction was 74 and 79%, respectively. The presence of any one of these four variables increased the odds of developing HFpEF by 3–4-fold, and the presence of all four variables increased the odds of developing HFpEF by 154-fold relative to the absence of all these factors in patients with underlying diastolic dysfunction. Therefore, if an individual patient in our population had 2 or more of these factors, then ***P*** would be > 53% and this patient would be predicted to be in the group that progressed to heart failure.

### Hierarchical clustering of patients with subclinical diastolic dysfunction into Phenogroups

**Fig. 1:**
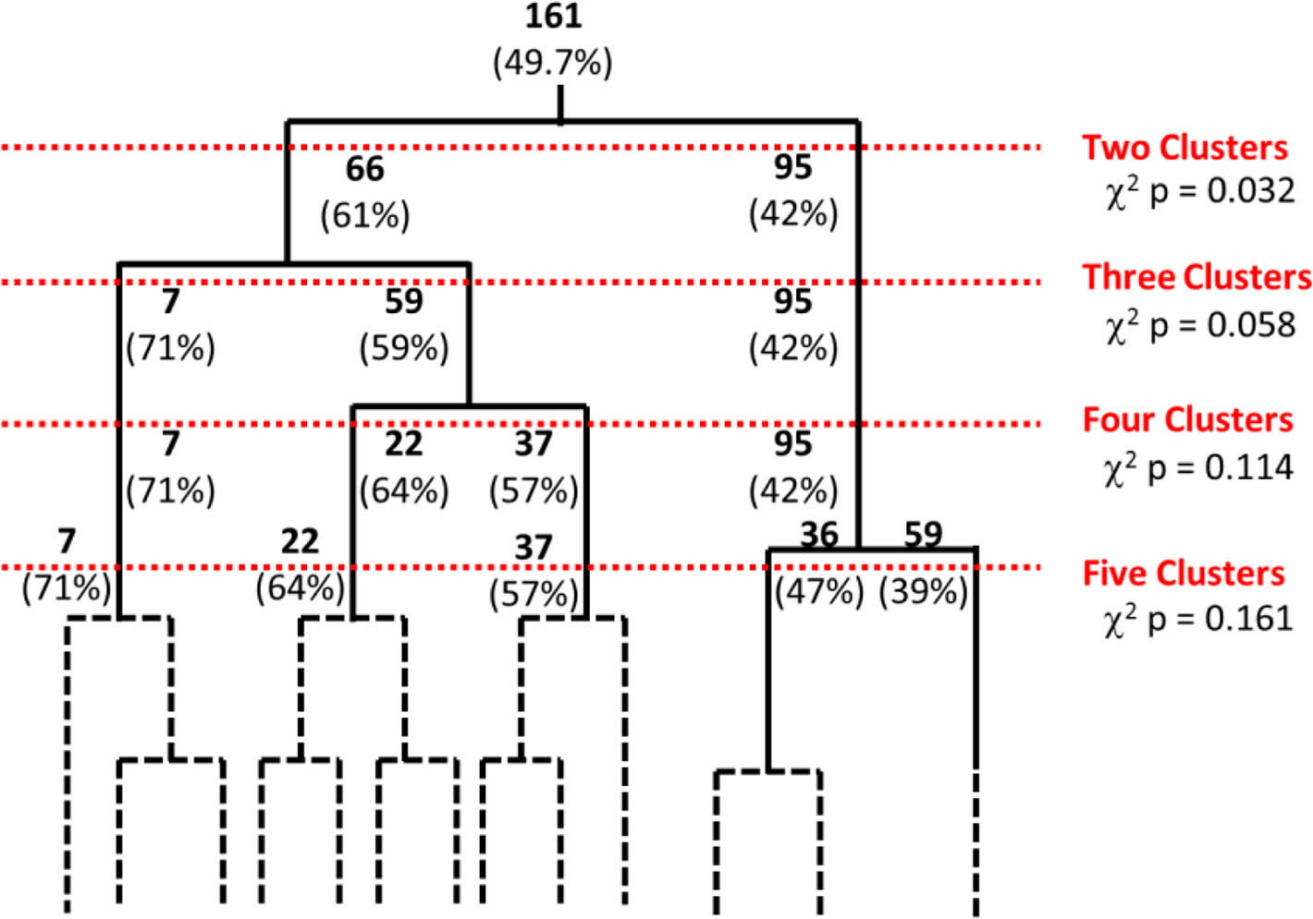
Diagram illustrating hierarchical clustering into 2, 3, 4 or 5 clusters. The original population of 161 asymptomatic diastolic dysfunction patients was repeatedly subdivided into smaller clusters based on phenotypic similarities with numbers in bold indicating the number of patients assigned to each cluster. Numbers in parentheses indicate the percentage of patients within the clusters who progress from diastolic dysfunction to HFpEF. Chi-squared *p* values indicate the probability of whether the frequency of HFpEF is the same among the clusters

After identifying the characteristics that predict the development of HFpEF in our cohort of patients with asymptomatic diastolic dysfunction, we then used unsupervised clustering to subdivide these patients into smaller groups with similar phenotypes. The goal was to examine the relationships between variables that group patients with similar phenotypes and which would then predict risk of developing HFpEF. Using the 65 variables (Supplemental Table 1) the 162 patients were subdivided using either hierarchical-based or density-based clustering analysis into various permutations of 2, 3, 4, and 5 groups, all with varying percentages of patients who developed HFpEF. With an increasing number of defined clusters, the larger clusters were effectively subdivided into smaller groups. Since there are no definitive criteria for determining the ideal number of clusters, we compared the percentages of patients in each cluster who developed clinical HFpEF, as a method of screening which clusters may have distinct phenotypes. We found the largest intergroup difference in proportion of patients that developed heart failure with the hierarchical 3-cluster grouping, which contained a high frequency HF group (71%), an intermediate frequency HF group (59%), and a low frequency HF group (42%) (*p* = 0.058) (Fig. 1). This grouping was chosen for further statistical analysis for the following reasons: [1] it contained a high frequency HFpEF group and a low frequency HFpEF group with the fewest number of clusters, [2] subdivision into further groups yielded non-significant differences in HF frequency among the clusters, [3] division into four clusters did not yield groups with higher or lower frequency of HF (only groups with intermediate frequency of HF), and [4] simplicity of analysis: fewer clusters would be more ideal for extracting statistically relevant conclusions due to sample size. Cluster purity for 3 hierarchical clusters was found to be 59%.

### Comparison of characteristics among phenogroups

Hierarchical clustering yielded three groups of patients with distinct phenotypic differences: Cluster A (*n* = 7); Cluster B (*n* = 59); and Cluster C (*n* = 95). The differences between these clusters are shown in Table 2. Variables which showed statistical differences (*p* < 0.05) are shown as well as those which approach significance (*p* < 0.10). When there was a difference among clusters, secondary analysis was used to determine which clusters differed from each other.

**Table 2:** Phenotypic Comparisons Among Clusters

	Cluster A (*n* = 7)	Cluster B (*n* = 59)	Cluster C (*n* = 95)	*P* value
**HFpEF %** (n)	**71.4%** (7)	**59.3%** (59)	**42.1%** (95)	0.058
Gender Male/Female (n)	4 / 3 (7)	28↑ / 31↓(59)	20↓ / 75↑ (95)	0.001
Chronic Kidney Disease (n)	(5)	(50)	(91)	0.031
Stage 1–2	0 ↓	18	44	
Stage 3a	1	8	23	
Stage 3b	3 ↑	10	13	
Stage 4	1	5	5	
Stage 5a	0	9 ↑	6	
NT-Pro B-type natriuretic peptide, pg/ml (n)	22,211 ± 63,387 (3)	13,816 ± 7801 (26)	3850 ± 2027 (42)	0.035§
**Echocardiography** Two-Dimensional Measurements				
LV posterior wall in diastole, cm (n)	1.37 ± 0.38 (7)	1.27 ± 0.06 (59)	1.06 ± 0.04 (95)	< 0.001*
LV posterior wall in systole, cm (n)	2.00 ± 0.48 (7)	1.91 ± 0.09 (58)	1.61 ± 0.05 (89)	< 0.001*
Ventricular septal wall diastole, cm (n)	1.44 ± 0.35 (7)	1.28 ± 0.06 (59)	1.1 ± 0.04 (95)	< 0.001*
LV mass, g (n)	276.09 ± 104.71 (7)	236.22 ± 18.15 (56)	162.75 ± 8.21 (89)	< 0.001*
LV mass index, g/m^2^ (n)	142.97 ± 44.83 (7)	114.72 ± 8.89 (56)	86.87 ± 4.07 (89)	< 0.001†
Cardiac Hypertrophy Severity (n)	(7)	(56)	(89)	< 0.001
None (count, %)	0 0.0% ↓	23 41.1% ↓	67 75.3% ↑	
Mild (count, %)	3 42.9%	9 16.1%	14 15.7%	
Moderate (count, %)	1 14.3%	8 14.3%	5 5.6%	
Severe (count, %)	3 42.9% ↑	16 28.6% ↑	3 3.4% ↓	
Fractional shortening, % (n)	41.44 ± 11.63 (7)	34.34 ± 2.77 (57)	33.33 ± 1.83 (93)	0.027‡
LV ejection fraction, % (n)	65.6 ± 8.08 (6)	60.23 ± 1.9 (49)	60.68 ± 1.18 (77)	0.024||
LV end-diastolic volume, mL (n)	133.09 ± 48.09 (6)	97.77 ± 10.17 (50)	75.41 ± 5.19 (81)	< 0.001†
End-diastolic volume index, mL/m^2^ (n)	70.35 ± 18.22 (6)	47.26 ± 4.61 (50)	40.23 ± 2.45 (81)	< 0.001†
LV end-systolic volume, mL (n)	45.79 ± 23.32 (7)	40 ± 4.46 (49)	29.94 ± 2.41 (79)	0.003*
End-systolic volume index, mL/m^2^ (n)	23.75 ± 10.06 (7)	19.30 ± 1.94 (49)	15.93 ± 1.14 (79)	0.001 *
Relative Wall thickness (n)	0.60 ± 0.19 (7)	0.55 ± 0.04 (58)	0.49 ± 0.02 (95)	0.080
Left atrium linear dimension, cm (n)	4.24 ± 0.9 (7)	4.24 ± 0.16 (59)	3.80 ± 0.11 (94)	0.044 #
Doppler Measurements				
Mitral peak E velocity, cm/s (n)	118.02 ± 17.42 (7)	111.79 ± 8.29 (56)	98.4 ± 4.8 (95)	0.081
Mitral peak A velocity, cm/s (n)	102.83 ± 40.87 (6)	89.7 ± 9.51 (55)	78.75 ± 4.58 (94)	0.064
Stroke volume LVOT, mL (n)	97.19 ± 35.38 (7)	83.97 ± 5.34 (28)	72.05 ± 5.73 (35)	0.003*
Stoke volume index LVOT, mL/m^2^ (n)	52.56 ± 20.84 (7)	41.61 ± 3.24 (26)	37.87 ± 3.23 (35)	0.002**
LVOT velocity max, cm/s (n)	137.59 ± 37.81 (7)	114.64 ± 5.67 (59)	103.51 ± 3.43 (94)	< 0.001†
LVOT velocity mean, cm/s (n)	95.76 ± 25.68 (7)	78.75 ± 3.96 (59)	70.24 ± 2.22 (93)	< 0.001†
LVOT max gradient, mmHg (n)	8.15 ± 3.83 (7)	5.53 ± 0.53 (57)	4.39 ± 0.29 (87)	< 0.001†
LVOT mean gradient, mmHg (n)	4.39 ± 1.91 (7)	2.93 ± 0.28 (57)	2.28 ± 0.14 (87)	< 0.001†
Aortic valve velocity max, cm/s (n)	364.35 ± 58.11 (7)	215.16 ± 16.83 (59)	174.47 ± 8.48 (94)	< 0.001†
Aortic valve velocity mean, cm/s (n)	243.57 ± 48.05 (7)	149.42 ± 12.01 (59)	118.26 ± 5.39 (93)	< 0.001†
Aortic valve max gradient, mmHg (n)	55.99 ± 18.79 (7)	20.17 ± 3.06 (59)	12.97 ± 1.26 (93)	< 0.001†
Aortic valve mean gradient, mmHg (n)	28.18 ± 10.8 (7)	10.9 ± 1.71 (59)	6.63 ± 0.61 (93)	< 0.001†

Categorical values are presented as counts and percentages; continuous variables are presented as mean ± 95% confidence interval. *LV* left ventricle, *LVOT* LV outflow tract

n = total number of patients in each cohort or n = number of patients with available data

↑ or ↓indicates higher or lower than expected by chance

*Clusters A & B differ from Cluster C (Newman-Keuls/Duncan’s)

† All Clusters differ from each other (Newman-Keuls)

‡ Only Cluster A differs from Cluster C (Duncan’s)

|| Only Cluster A differs from Cluster B (Duncan’s)

# ANOVA is significant but multiple comparisons (Newman-Keuls and Duncan’s) show all clusters overlap each other

** Clusters B & C differ from Cluster A (Newman-Keuls)

Cluster A (*n* = 7) had the highest frequency of patients with subclinical diastolic dysfunction who progressed to HFpEF (71.4%) and the lowest percentage of females (42.9%). This cluster was characterized as having severe cardiac hypertrophy and moderate aortic stenosis. All the patients in this group had some degree of cardiac hypertrophy (mild, moderate, or severe), with significantly more patients having severe cardiac hypertrophy than expected. In addition, Cluster A patients tended to have the highest NT-proBNP and the highest LV systolic function as determined by ejection fraction, fractional shortening, and stroke volumes when compared to the other groups. Interpretation of results for Cluster A may be unreliable due to the small size of this cluster and are presented for comparative purposes only.

Cluster B (*n* = 59) had an intermediate frequency of patients with diastolic dysfunction who progressed to HFpEF at 59.3%. In this group 52.5% were female, and 47.5% were male. These patients tended to be taller, heavier, and with the largest body surface area (*p* < 0.10 for each). They had mild to moderate cardiac hypertrophy and mild aortic stenosis. 58.9% of patients had some degree of cardiac hypertrophy, with significantly more patients having severe cardiac hypertrophy and fewer patients with no cardiac hypertrophy than expected. This cluster averaged mid-range NT-proBNP levels and had more patients with severe CKD.

Cluster C (*n* = 95) had the lowest frequency of patients with diastolic dysfunction that developed HFpEF (42.1%) and was comprised mostly of females (78.9%) who tended to be physically smaller than those patients in Cluster B based on height, weight, and BSA (*p* < 0.10 for each). This group, on average, had neither cardiac hypertrophy nor aortic stenosis. Of these patients, only 25% had some degree of cardiac hypertrophy, with fewer patients than expected having severe cardiac hypertrophy. NT-proBNP levels were the lowest in this group and patients overall had milder stages of CKD. This group still had preserved LV systolic function but closer to the lower limits of normal based on fractional shortening, LV volumes and stroke volumes.

### Intracluster analysis of patients who develop HFpEF vs. those who remain in asymptomatic diastolic dysfunction

Each of the 3 clusters contained patients who developed HFpEF and those who remained asymptomatic. Therefore, we analyzed which variables significantly differed between outcomes within each cluster (Table 3). Some variables distinguish those who remained asymptomatic from those who progressed to HFpEF in only one of the clusters whereas other factors distinguish those who remained asymptomatic from those who progressed in multiple clusters.

**Table 3:** Differences in variables between the two outcomes of developing HFpEF versus remaining asymptomatic within each cluster

**Cluster A**			
	Subclinical DD (*n* = 2)	HFpEF (*n* = 5)	*p* value*
Aortic distensibility	0.00293 ± 0.00773 (*n* = 2)	0.00103 ± 0.00062 (*n* = 4)	0.016
**Cluster B**			
	Subclinical DD (*n* = 24)	HFpEF (*n* = 35)	*p* value*
**Co-morbidities**			
Chronic kidney disease^||^	27% (*n* = 22)	69% (*n* = 35)	0.006
Diabetes	27% (*n* = 22)	66% (*n* = 35)	0.011
**Medication Use by Class**			
Aldosterone antagonists	29% (*n* = 21)	6% (*n* = 35)	0.048
Beta blockers	48% (*n* = 21)	80% (*n* = 35)	0.026
Diuretics^||^	29% (*n* = 21)	60% (*n* = 35)	0.045
**Echocardiographic Parameters**			
LV Internal dimension in systolic (cm)	2.84 ± 0.28 (*n* = 23)	3.23 ± 0.23 (*n* = 35)	0.036
LV Outflow Tract max gradient (mmHg)	5.8 ± 0.86 (*n* = 23)	5.34 ± 0.69 (*n* = 34)	0.049
Aortic Valve velocity max (cm/sec)	235.82 ± 31.84 (*n* = 24)	201 ± 18.04 (*n* = 35)	0.041
Aortic Valve velocity mean (cm/sec)	163.64 ± 22.82 (*n* = 24)	139.68 ± 12.89 (*n* = 35)	0.049
Aortic Valve max gradient (mmHg)	24.43 ± 5.98 (*n* = 24)	17.24 ± 3.03 (*n* = 35)	0.020
Aortic Valve mean gradient (mmHg)	13.22 ± 3.39 (*n* = 24)	9.32 ± 1.67 (*n* = 35)	0.024
**Vascular parameters**			
Pulse pressure (mmHg)	65.57 ± 6.49 (*n* = 14)	82.79 ± 9.87 (*n* = 18)	0.004
Arterial stiffness (mmHg/mL/m^2^)	1.61 ± 0.3 (*n* = 11)	2.24 ± 0.42 (*n* = 14)	0.013
Arterial elastance (mmHg/mL)	1.47 ± 0.21 (*n* = 11)	1.87 ± 0.28 (*n* = 14)	0.021
Aortic distensibility (1/mmHg)	0.00291 ± 0.00088 (*n* = 14)	0.00189 ± 0.00059 (*n* = 18)	0.047
**Cluster C**			
	Subclinical DD (*n* = 55)	HFpEF (*n* = 40)	*p* value*
**Co-morbidities**			
Atrial fibrillation	19% (*n* = 52)	45% (*n* = 40)	0.015
Chronic kidney disease^||^	25% (*n* = 52)	55% (*n* = 40)	0.007
Coronary artery disease	40% (*n* = 52)	65% (*n* = 40)	0.033
**Medication use**			
Digoxin	0% (*n* = 51)	13% (*n* = 40)	0.033
Diuretics^||^	38% (*n* = 50)	73%(*n* = 40)	0.002
**Echocardiographic Parameters**			
LV Posterior Wall in Systole (cm)	1.56 ± 0.06 (*n* = 50)	1.67 ± 0.1 (*n* = 39)	0.041
LV End-diastolic volume (mL)	80.37 ± 7.41 (*n* = 48)	68.2 ± 6.47 (*n* = 33)	0.021
End-diastolic volume index (mL/m^2^)	42.78 ± 3.44 (*n* = 48)	36.52 ± 3.14 (*n* = 33)	0.012
LV End-systolic volume (mL)	32.12 ± 3.37 (*n* = 48)	26.56 ± 3.08 (*n* = 31)	0.024
End-systolic volume index (mL/m^2^)	17.06 ± 1.57 (*n* = 48)	14.19 ± 1.47 (*n* = 31)	0.013
**Vascular parameters**			
Diastolic blood pressure (mmHg)	75.85 ± 3.37 (*n* = 33)	68.82 ± 6.34 (*n* = 17)	0.030

Only variables which differed between the subclinical diastolic dysfunction (DD) group and group that progressed to HFpEF are shown

Categorical values are presented as counts and percentages; continuous variables are presented as mean ± 95% confidence interval; *p value = asymptomatic vs. HFpEF outcome

n = total number of patients in each cohort or n = number of patients with available data; || Chronic kidney disease and diuretics differ between Subclinical DD and HFpEF groups in more than one cluster

Within cluster A, decreased aortic distensibility was seen in the group that progressed to HFpEF. In patients within cluster B, chronic kidney disease, diabetes and use of beta blockers and diuretics were seen in the group that developed HFpEF. These patients also had lower values for LVOT max gradient, and lower velocities and gradients across the aortic valves as well as decreased aortic distensibility. They had increased LV internal dimension, a higher pulse pressure, increased arterial stiffness, and increased arterial elastance. Patients in cluster B who remained asymptomatic were more likely to be taking aldosterone antagonists. Patients within cluster C who developed HFpEF were more likely to have a history of chronic kidney disease, coronary artery disease, atrial fibrillation, digoxin and diuretics use. They also were more likely to have echocardiographic parameters consistent with increased systolic LV posterior wall thickness (LVPWs) and decreased LV systolic and diastolic volumes (LVESV and ESVI, and LVEDV and EDVI) along with lower diastolic blood pressure.

Within each cluster, logistic regression was used to identify which factors were significant and independent predictors of those who remain in asymptomatic diastolic dysfunction and those who progressed to HFpEF.

There were too few patients in Cluster A to determine variables which were significant predictors of HFpEF via logistic regression. In Cluster B, diabetes, chronic kidney disease, diuretics use, aortic valve (Ao V2) max gradient (in mmHg), and diastolic wall strain (as fraction) were found to be independent predictors of progression to HFpEF while adjusting for other factors, (SN/SP 76.5/71.4%, at cutoff of ***P*** = 61.8% representing HFpEF frequency for the 55 of 59 patients with complete data for these variables).

$$ \boldsymbol{P}=\frac{\mathbf{1}}{\mathbf{1}+{\boldsymbol{e}}^{\left(\mathbf{4.44}\hbox{-} {\left(\mathbf{0}\kern0.5em \boldsymbol{or}\kern0.5em \mathbf{2}.\mathbf{30}\right)}_{\boldsymbol{DM}}\hbox{-} {\left(\mathbf{0}\kern0.5em \boldsymbol{or}\kern0.5em \mathbf{2}.\mathbf{30}\right)}_{\boldsymbol{CKD}}\hbox{-} {\left(\mathbf{0}\kern0.5em \boldsymbol{or}\kern0.5em \mathbf{2}.\mathbf{28}\right)}_{\boldsymbol{Diuretic}}+\mathbf{0.11}\ast \boldsymbol{Ao}\kern0.5em \boldsymbol{V}\mathbf{2}\kern0.5em \boldsymbol{\max}\kern0.5em \boldsymbol{gradient}\hbox{-} \mathbf{13.29}\ast \boldsymbol{Diastolic}\kern0.5em \boldsymbol{wall}\kern0.5em \boldsymbol{strain}\right)}} $$

In Cluster C, independent predictors of progression to HFpEF were found to be chronic kidney disease, diuretics use, age at diagnosis of diastolic dysfunction, and indexed end-systolic volume (ESVI) while adjusting for other factors; (SN/SP = 80.6/77.3%, at cutoff of ***P*** = 41.3% representing HFpEF frequency for the 75 of 95 patients with complete data for these variables).

$$ \boldsymbol{P}=\frac{\mathbf{1}}{\mathbf{1}+{\boldsymbol{e}}^{\left(\mathbf{3.12}-{\left(\mathbf{0}\ \boldsymbol{or}\ \mathbf{1.23}\right)}_{\boldsymbol{CKD}}-{\left(\mathbf{0}\ \boldsymbol{or}\ \mathbf{1.79}\right)}_{\boldsymbol{Diuretic}}-\mathbf{0.06}\ast \boldsymbol{Age}+\mathbf{0.17}\ast \boldsymbol{ESVI}\right)}} $$

### Two-fold cross validation of the HFpEF outcome predictive modeling (supplementary table 2)

All predictions for HFpEF outcome, based on a discovery cohort and then applied to both the discovery and validation cohorts, were greater than a random outcome of 50%. In general, the predictions for the discovery cohorts were slightly higher than the predictions for the validation cohorts. The lowest predictions were found when optimum predictive parameters for cluster B were applied to cluster C and vice versa. Within the same population (entire group, cluster B or cluster C) the discovery cohort can reasonably predict HFpEF outcome for the validation cohort. However, using cluster B population to predict cluster C population (or vice versa) yields poorer predictions. This indicates that the predictions should be used for populations with similar characteristics to that used for deriving the predictions.

### Kaplan-Meier estimates of events

**Fig. 2:**
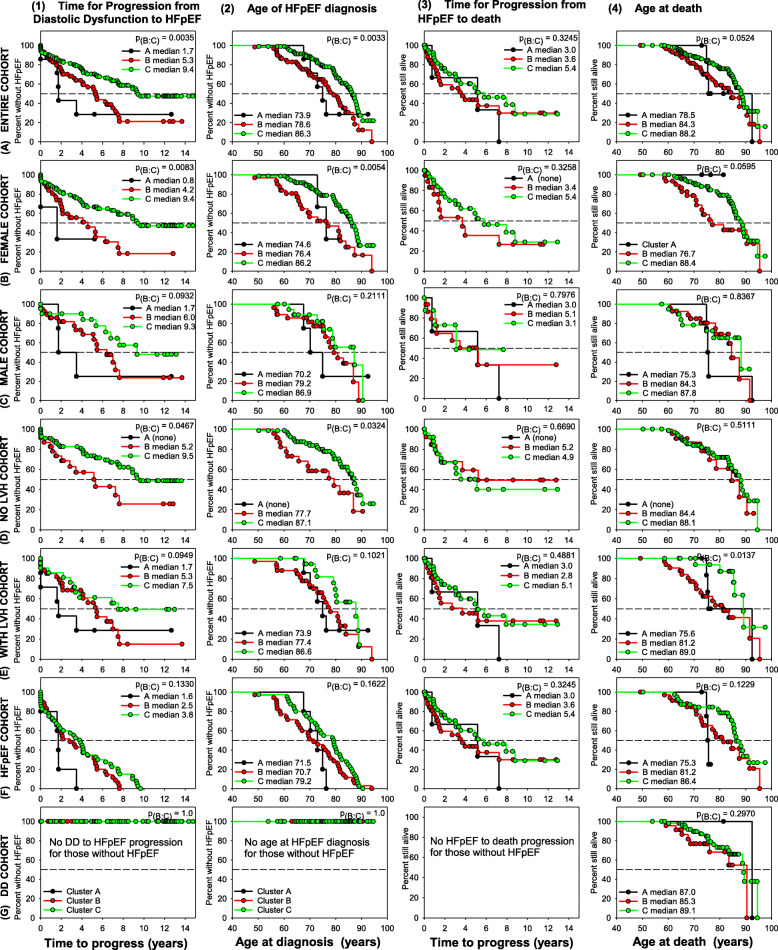
Summary of significant Kaplan-Meier differences among Clusters. Kaplan-Meier estimates for the (**1**) time (years) from the initial diagnosis of subclinical diastolic dysfunction (DD) to HFpEF (left column), (**2**) age of HFpEF diagnosis, (**3**) time (years) from the development of HFpEF to death from all causes, and (**4**) age of death (right column). Each graph shows comparisons among the three Clusters A (black), B (red), and C (green). Kaplan-Meier analysis was performed on the (**A**) entire cohort, (**B**) female only cohort (**C**) male only cohort (**D**) all patients without left ventricular (LV) hypertrophy (**E**) all patients with LV hypertrophy (**F**) all patients who developed HFpEF and (**G**) all patients who remained with subclinical diastolic dysfunction (DD). *P* values = Cluster B versus Cluster C

Kaplan-Meier analysis was performed to compare the clusters for different time to events. When comparing among the three clusters, differences were found and are shown in Fig. 2. The small sample size of Cluster A may limit the significance of the findings from this cluster. No significant within-cluster differences were found when stratified by gender, presence or absence of LVH, or for remaining asymptomatic vs. progression to HFpEF.

Patients in Clusters A, B, and C developed diastolic dysfunction at similar ages (Cluster A: median age 71.6 years, Cluster B: median age 67.9 years, and Cluster C: median age 72.9 years; Cluster B vs. C, *p* = 0.5712). There were no differences in age of diastolic dysfunction diagnosis when stratifying each cluster by gender, by presence of LVH, or by HFpEF outcome (data not shown). The three clusters progressed from diastolic dysfunction to HFpEF at different rates (Fig. 2**, A1**). Cluster A progressed to HFpEF the fastest (median 1.7 years), Cluster B progressed at an intermediate rate (median 5.3 years), and Cluster C progressed the slowest (median 9.4 years; Cluster B vs. C, *p* = 0.0035). The same trend was seen when the patients from each cluster were stratified by gender. Females in Cluster B develop HFpEF in a statistically shorter timeframe than females in Cluster C (Fig. 2**, B1**) whereas the males in Clusters B and C show no statistical difference in time interval of developing HFpEF (Fig. 2**, C1**).

All patients in Cluster A had some degree of LVH, thus comparison among the clusters based on the absence or presence of LVH (Fig. 2**, D and E**) is applicable only to Clusters B and C. Patients in clusters B developed HFpEF at the same rate whether or not they had LVH. Only those without LVH showed a difference between clusters B and C (Fig. 2**, D1 and E1**). When the patients who are known to develop HFpEF are separately analyzed for the time interval of progressing from asymptomatic diastolic dysfunction to HFpEF, there is no significant differences whether they are in Cluster A, B or C (Fig. 2**, F1 and G1**).

Clusters also differed in age of diagnosis of HFpEF (Fig. 2**, A2**). Cluster A developed HFpEF at the youngest age (median age 73.9 years), Cluster B at an intermediate age (median age 78.6 years), and Cluster C the oldest (median age 86.3 years; Cluster B vs. C, *p* = 0.0033). Figure 2**, B2** shows statistically significant differences between females in Cluster B vs. Cluster C which were not seen in the male cohort (Fig. 2**, C2**). Patients without LVH differed in age of HFpEF diagnosis depending on whether they were in Cluster B or Cluster C. This contrasts with the patients who had LVH (Fig. 2**, E2**) who had a non-significant median difference in age when diagnosed with HFpEF. In general, when considering only those who progress to HFpEF, the age at HFpEF diagnosis does not differ significantly between the three clusters (Fig. 2**, F2**). None of the patients with diastolic dysfunction who remained asymptomatic throughout the duration of the study developed HFpEF and Fig. 2**, G2**.

The three clusters differed significantly in age at death **(**Fig. 2**, A4**). Patients in Cluster A died at the youngest age (median age 78.5 years), Cluster B at an intermediate age (median age 84.3 years), and cluster C at the oldest age (median age 88.2 years; Cluster B vs. C, *p* = 0.0524). When stratifying by gender, (Fig. 2**, B4 and C4**), females in Cluster B died at a younger age than females in Cluster C (median age of death 76.7 years vs. 88.4 years, *p* = 0.0595). Males in Cluster B and Cluster C did not differ and had similar median ages of death when compared to the entire cohort (males + females). In addition, when stratifying by the absence or presence of LVH (Fig. 2**, D4 and E4**), there was no difference in age at death between Cluster B and Cluster C for patients without LVH. In contrast, those patients with LVH in Cluster B died at a younger age than those in Cluster C (median age of death 81.2 years vs. 89.0 years, *p* = 0.0137).

The time to progress from first being diagnosed with HFpEF to death is only applicable to those patients who developed HFpEF. The median time for progression from HFpEF to death due to all causes for Cluster A patients was 3.0 years, for Cluster B was 3.6 years, and for Cluster C was 5.4 years; Cluster B vs. C, *p* = 0.3245 (Fig. 2**, A3**). The time interval between diagnosis of HFpEF and all-cause death did not differ when stratifying the clusters by gender (Fig. 2**, B3 and C3)** or LVH (Fig. 2**, D3 and E3**). This suggests that survival time after diagnosis of HFpEF is independent of any differences in associated comorbidities.

## Discussion

In this retrospective study of 162 patients with asymptomatic diastolic dysfunction who were matched by known outcome (remaining asymptomatic vs. developing HFpEF), we used unsupervised machine learning to determine whether patients could be clustered into groups of similar phenotypes which would be predictive of developing HFpEF. In this process, we found (1) patients with asymptomatic diastolic dysfunction were a heterogeneous group with different risk profiles (2) all clusters contained some patients who would progress to develop HFpEF (3) the identified phenogroups had differential outcomes, indicating different risk profiles and clinical trajectories (4) certain co-morbidities were risk factors for developing HFpEF across the entire cohort and other risk factors for developing HFpEF were restricted to specific clusters/phenogroups and (5) the time interval between developing HFpEF and death was similar regardless of cluster assignment (data not shown).to This study showed that it was feasible to subdivide asymptomatic diastolic dysfunction patients, who have not yet progressed to clinical HFpEF, early in the disease process into smaller more homogeneous phenogroups with well-defined risk factors for progression to HFpEF and different clinical trajectories. This approach may be useful to identify those patients with subclinical diastolic dysfunction who are at higher risk and who may benefit from tailored preventive strategies.

Our population of patients with subclinical diastolic dysfunction has similar demographic features and comorbidities compared to patients with HFpEF [2–6, 16, 17]. Compared to the Meta-analysis Global Group in Chronic Heart Failure, our patients were also older when diagnosed with HFpEF (74 vs. 71 years old), are more often female (68% vs. 50%), and have a higher prevalence of multiple systemic pro-inflammatory non-cardiac comorbidities including obesity or metabolic syndrome, HTN, DM, and CKD [16].

To the best of our knowledge, our study is the first analysis to evaluate clinical characteristics through logistic regression in order examine predictors of HFpEF in patients with subclinical diastolic dysfunction. One strength of our study was to use a retrospective data set in which the outcomes of progressionto clinical HFpEF were known, but clinical and echocardiographic data prior to this progression were available for analysis and allowed for matching of patients with asymptomatic diastolic dysfunction but different outcomes.

For our entire cohort, a history of diabetes, CKD, atrial fibrillation, and diuretic use were found to be independent positive predictors of progression from subclinical diastolic dysfunction to HFpEF. HTN was the most prevalent co-morbidity in our population of patients with subclinical diastolic dysfunction. Therefore, although HTN is not a discriminating predictor of which patients will develop HFpEF, it is a common risk factor for the development of both diastolic dysfunction and HFpEF.

It has been suggested that the pathophysiology of the development of diastolic dysfunction and progression to HFpEF is closely linked to systemic inflammation from the presence of the non-cardiac comorbidities (obesity/metabolic syndrome, HTN, DM, CKD, anemia, COPD) [3]. Increased levels of the proinflammatory cytokines IL-1β and IL-12 have been shown to be associated with HFpEF [18]*.* Chronic systemic inflammation causes coronary microvascular endothelial inflammation, leading to interstitial fibrosis and cardiac hypertrophy [2, 3], which ultimately leads to diastolic dysfunction and development of HFpEF. Recently, Spiesshoefer et al. 2019, have speculated that respiratory muscle function may be affected in HFpEF by inflammatory markers [19], however these were not measured in our study. Arterial HTN is the most prevalent comorbidity in HFpEF, and is thought to induce HFpEF through microvascular inflammation [3]. This is in contrast to prior paradigms of HFpEF that suggested longstanding HTN caused HFpEF through myocardial afterload excess. This is also distinctly separate from the mechanism of eccentric LV remodeling and dilatation in HFrEF that is caused by cardiomyocyte loss from ischemia, infection, and toxicity [3]. Hence, HFpEF is more likely to be a non-ischemic cardiomyopathy, whereas HFrEF is more likely ischemic in etiology. These systemic non-cardiac comorbidities that are believed to contribute to HFpEF are the same comorbidities found to be predictive of HFpEF in our population, and more prevalent in the cohort that progressed to HFpEF. This may be further evidence that early recognition, diagnosis and treatment of these comorbidities may delay or prevent disease progression.

Our study was also unique by using logistic regression to identify variables that were independent positive predictors for the development of HFpEF within each cluster. Predictions for clusters are more specific than those for the entire population since clusters represent patients with similar features. Predictions are tailored for the specific phenotype of each cluster. Once the three clusters were identified, the variables that remained independent positive predictors for development of HFpEF were CKD and diuretic use in Clusters B and C, and diabetes in Cluster B. In patients with subclinical diastolic dysfunction, early recognition and treatment of diabetes, CKD, and HTN may delay progression to clinical HFpEF, and presence of these comorbidities as well as atrial fibrillation and diuretic requirement may be used to prognosticate the risk of disease progression.

Despite the lower risk of death in HFpEF (regardless of age, gender, and etiology of HF) compared to patients with HFrEF, those with HFpEF still have high absolute mortality, and unfortunately do not benefit from neurohormonal antagonists (i.e. beta blockers, angiontensin converting enzyme (ACE) inhibitors, angiotensin receptor blockers (ARBs), mineralocorticoid receptor antagonists, ARB-neprilysin inhibitors) or intracardiac devices as well as do patients with HFrEF [2, 4, 5, 16]. Given the lack of standardized and effective therapies for treatment of diastolic dysfunction and HFpEF, the subclassification into smaller homogeneous subgroups of patients with diastolic dysfunction at risk for developing HFpEF may be the first step to conduct future studies that look at phenotypic differences in response to medical therapy, which could further lead to individualized treatment and improved prognosis of the disease.

Other investigators have used machine learning or cluster analysis to identify subgroups of patients with distinct phenotypes that differ in their risk profiles and survival outcomes [7–11]. These researchers have studied heterogeneous populations of patients with primary HTN and the absence of HF [9], HFpEF alone [7, 8], and mixed populations of HFrEF and HFpEF combined [10, 11]. However, to our knowledge, ours is the first study to use hierarchical clustering to identify subgroups of patients with subclinical diastolic dysfunction. Clustering of our cohort of patients resulted in three groups with distinct phenotypes and different disease trajectories and prognosis. Cluster A was a smaller (*n* = 7) high-risk group with the highest frequency of HFpEF, lowest percentage of females, presence of severe cardiac hypertrophy, moderate AS, and highest NT-proBNP. The group size was not powered to reach statistical significance when comparing survival analysis with other clusters, though based on trends we suspect that this group would have the poorest disease trajectory. Cluster B (*n* = 59) was a moderate-risk group with an intermediate frequency of HFpEF, intermediate percentage of females, presence of mild to moderate cardiac hypertrophy, mild AS, mid-range NT-proBNP, and more severe CKD. This cluster did more poorly than cluster C in terms of shorter time to progress from diastolic dysfunction to HFpEF, younger age at diagnosis of HFpEF, and age of death. Cluster C (*n* = 95) was a low-risk group with the lowest prevalence of HFpEF, highest percentage of females, no cardiac hypertrophy, no AS, lowest NT-proBNP, and milder CKD stages. This cluster had the best prognosis in terms of disease progression. The primary reason for Cluster C having an overall slower progression from diastolic dysfunction to HFpEF and later age at HFpEF diagnosis is due to this group having a larger percentage of patients that never progress to HFpEF; for those patients who do progress in Cluster B and Cluster C, time to progression and age at diagnosis of HFpEF was not different. In general, cluster differences regarding survival curve disease trajectories were likely due to global phenotypic differences between these clusters, rather than any distinguishing variable. Gender did not contribute to rate of disease progression, and the outcome of whether patients developed HFpEF was not related to age at diagnosis of diastolic dysfunction or age at death.

Katz et al. [9] used hierarchical clustering to divide 1273 patients with primary HTN into two clinically distinct subgroups, in order to study the phenotypes of patients who might be at higher risk for developing HFpEF, although this outcome was never followed. Common comorbidities included obesity, diabetes, CAD, and CKD, with several of these being more prevalent in the higher-risk group, which is similar to our findings. On average, both groups had an elevated *E/e’* ratio suggesting diastolic dysfunction, though diastolic dysfunction was not specifically evaluated and may not have been present in all patients; patients also had zero to mild LVH based on LVMI. This population may be the most similar to our study population of patients with subclinical diastolic dysfunction, although it is difficult to compare their results to ours given different variables used to define phenogroups, and that survival analysis was not conducted.

Shah et al. [7] used hierarchical clustering to subdivide 397 patients with HFpEF into three phenotypically distinct subgroups that differed in risk profiles based on outcomes of cardiovascular or heart failure hospitalization and death. The highest-risk group had the highest prevalence of atrial fibrillation and CKD, and highest NT-proBNP whereas the moderate-risk group had the highest prevalence of diabetes. This is similar to the analysis done by Kao et al. [8] which used latent class analysis to divide 4113 patients with HFpEF enrolled in the I-PRESERVE (Irbesartan in Heart Failure with Preserved Ejection Fraction) study into 6 phenotypically distinct subgroups differing in all-cause mortality and cardiovascular hospitalization outcomes. The two highest-risk subgroups were characterized by a high prevalence of atrial fibrillation, CKD, diabetes and obesity. In our study of patients with subclinical diastolic dysfunction, the presence of atrial fibrillation, CKD, and diabetes were predictive of progression to HFpEF in the entire cohort regardless of clustering results. We also found that between clusters, NT-proBNP levels were correlated with the frequency of patients who progressed from subclinical diastolic dysfunction to HFpEF. Our study did not look at obesity but matched patients for BSA which would decrease the contribution of BSA to HFpEF progression.

Ahmad et al. [10] and Horiuchi et al. [11] both used K-means clustering to analyze a mixed population of HFrEF and HFpEF. Ahmad et al. [10] subdivided a larger population of 44,886 patients from the Swedish Heart Failure Registry into 4 subgroups that differed significantly in terms of 1-year survival and response to medication class (diuretics, ACE-Inhibitors, beta blockers, and nitrates). Their cluster with the largest percentage of patients with preserved EF > 50% shared features similar to our population, but in this cluster only 34% of patients had HFpEF. In addition, The Ahmad et al. [10] study grouped the entire population a second time by LVEF values, and those patients with LVEF > 50% again shared these features—older age, female predominance, more likely to have a non-ischemic cause of cardiomyopathy, and a high prevalence of comorbidities (HTN, atrial fibrillation, CKD, aortic stenosis, and diabetes). We did not include malignancy, anemia, and COPD in our data sets which were included in the analysis from the Swedish Heart Failure Registry. These patients were least often treated with neurohormonal therapies (beta blockers, ACE-Inhibitors) and implanted device therapies (ICD, cardiac resynchronization therapy-defibrillator). Horiuchi et al. [11] studied a smaller population of 345 consecutively admitted patients with acute heart failure hospitalized in the cardiovascular intensive care unit, with similar findings.

In brief, the previous studies [7–11] subclassified their patient populations into smaller phenotypically distinct groups with unique clinical trajectories in terms of outcomes and response to various treatments. Direct comparison with our results is difficult due to the differences in initial patient population, the variables available and used for clustering, the distinguishing variables that define each phenogroup, and the variation in outcome measures used to risk-stratify phenogroups and report survival analysis.

### Limitations

This is a retrospective study with a relatively small cohort with some data being incomplete. Missing data points had to be imputed using a singular value decomposition technique in order to be able to perform the clustering. Analysis of the clusters however used only the available data. Because of the small study population, we were unable to a priori divide the population into a test cohort and a validation cohort. In this study we determined predictors for progression from diastolic dysfunction to HFpEF based on a population in which 50% of the patients progressed. The coefficients for the logistic regressions would not apply to other populations and would depend on the HFpEF prevalence in the population being studied. Thus, the applicability of cluster analysis to clinical practice is limited at this time. However, the variables which we found to be correlated with, and predictive of HFpEF outcome, are likely to be similar to those that would be found for other populations. Levels of inflammatory cytokines, which may play a role in the development of HFpEF were not available for this study.

## Summary and conclusion

We have shown that cluster analysis can separate patients with diastolic dysfunction into different phenotypic subgroups which differ in HFpEF and mortality outcomes and have different variables correlated with and predictive of HFpEF outcome. Our findings may be applicable to other populations, as the characteristics of our patients with diastolic dysfunction are similar to those described in the literature for patients with HFpEF. Confirmation of these findings could be done using a validation cohort of patients with diastolic dysfunction who could be sorted into our pre-defined clusters using supervised machine learning, and their characteristics and disease trajectories compared to those of our study population.

Future clinical trials may be designed to evaluate response to therapies in phenotypically different subpopulations of patients with diastolic dysfunction or HFpEF. Additionally, future clinical trials may also be designed to study prevention of development of HFpEF in patients with diastolic dysfunction. Cluster analysis may be useful to indicate early in the disease process which patients are at highest risk of progressing to clinical heart failure, and may be an important first step in studying which therapy may lead to the best response for a particular phenogroup. Ultimately, we hope that this method will help identify high-risk patients and may help with selecting individualized treatment modalities that are effective in preventing disease progression and improving morbidity and mortality.

## Supplementary information

**Additional file 1: Table S1.** Variables used during clustering analysis.

**Additional file 2: Table S2.** Two-fold cross validation of the HFpEF outcome predictive modeling.

## Data Availability

The datasets used and /or analyzed during the current study are available from the corresponding author on reasonable request.

## Abbreviations

ANOVA: Analysis of variance
Ao: Aortic
AFib: Atrial fibrillation
CKD: Chronic kidney disease
DM: Diabetes mellitus
EDVI: End-diastolic volume index
ESVI: End-systolic volume index
EF: Ejection fraction
HFpEF: Heart failure with preserved ejection fraction
HTN: Hypertension
ICD-9: International classification of diseases, 9th edition
LV: Left ventricular
LVOT: Left ventricular outflow tract
LVEDV: LV end diastolic volume
LVESV: LV end systolic volume
NT-proBNP: N-terminal pro hormone BNP
TTEs: Transthoracic echocardiograms
SBP/DBP: Systolic and diastolic blood pressure
LVPWs: Systolic LV posterior wall thickness
