# Supplementary material for: Identifying Phenogroups in patients with subclinical diastolic dysfunction using unsupervised statistical learning
Source: BMC Cardiovasc Disord. 2020 Aug 14;20:367. doi: 10.1186/s12872-020-01620-z (PMC7427922; doi:10.1186/s12872-020-01620-z)
Supplement: Supplementary file 1 — Additional file 1: Table S1. Variables used during clustering analysis. [file 12872_2020_1620_MOESM1_ESM.docx]

| **Supplementary Table 1: Variables used during clustering analysis** | |
| --- | --- |
| **Demographics** | Age, gender, race |
| **Physical characteristics** | Height, weight, body surface area, systolic and diastolic blood pressure, and pulse pressure |
| **Clinical History** | hypertension, diabetes, chronic kidney disease, alcohol use, smoking, coronary artery disease, cardiovascular accident or transient ischemic attack, atrial fibrillation |
| **Medication use by class** | beta blockers, calcium channel blockers, angiotensin-converting-enzyme inhibitors, angiotensin II receptor blockers, digoxin, diuretics, aldosterone antagonists |
| **Clinical findings** | GFR, N-terminal prohormone of brain natriuretic peptide |
| **Echocardiography** |  |
| Left heart structure | LV end-diastolic volume, LV end-diastolic volume index, LV end-systolic volume, LV end-systolic volume index, LV end-diastolic dimension, LV end-systolic dimension, LV end-diastolic posterior wall thickness, LV end-systolic posterior wall thickness, end-diastolic interventricular septal thickness, LV mass, LV mass indexed, left atrial volume index, left atrial linear dimension, relative wall thickness, diastolic wall strain, atrioventricular coupling |
| LV systolic function | LV end-systolic pressure, LV ejection fraction, fractional shortening, LV contractility |
| LV diastolic function | Degree of diastolic dysfunction, Mitral inflow characteristics: mitral peak E velocity, mitral peak A velocity, E/A ratio |
| Hemodynamics | LV stroke volume, LV stroke volume index, LV outflow tract maximum velocity, outflow tract mean velocity, outflow tract maximum gradient, outflow tract mean gradient, Ao V2 maximum velocity, Ao V2 mean velocity, Ao V2 maximum gradient, Ao V2 mean gradient |
| **Aorta/Arterial compliance** | ascending aorta root diameter at end diastole, ascending aorta root diameter at end systole, Arterial stiffness, arterial elastance, aortic distensibility |
| LV = left ventricular, GFR = glomerular filtration rate | |
