# Supplementary material for: Identifying Phenogroups in patients with subclinical diastolic dysfunction using unsupervised statistical learning
Source: BMC Cardiovasc Disord. 2020 Aug 14;20:367. doi: 10.1186/s12872-020-01620-z (PMC7427922; doi:10.1186/s12872-020-01620-z)
Supplement: Supplementary file 2 — Additional file 2: Table S2. Two-fold cross validation of the HFpEF outcome predictive modeling. [file 12872_2020_1620_MOESM2_ESM.docx]

**Supplementary Table 2: Two-fold cross validation of the HFpEF outcome predictive modeling**

| Study subpopulation | * Logistic regression variables | Number with complete data | ** Number (%) progressed to HFpEF | % correctly categorized | Sensitivity & Specificity | P value compared to random prediction |
| --- | --- | --- | --- | --- | --- | --- |
| Entire population N=162 | Histories of diabetes, CKD, atrial fibrillation, and diuretic use |  |  |  |  |  |
| Discovery cohort = 1^st^ half |  | 78 of 81 | 42 (53.8%) | 77% | 79% / 75% | 0.000002 |
| Validation cohort = 2^nd^ half |  | 74 of 81 | 39 (52.7%) | 77% | 69% / 86% | <0.000001 |
|  |  |  |  |  |  |  |
| Discovery cohort = 2^nd^ half |  | 74 of 81 | 39 (52.7%) | 77% | 74% / 80% | <0.000001 |
| Validation cohort = 1^st^ half |  | 78 of 81 | 42 (53.8%) | 67% | 69% / 64% | 0.0032 |
|  |  |  |  |  |  |  |
| Cluster B  N=59 | Histories of diabetes, CKD, and diuretic use, AoV max gradient, diastolic wall strain |  |  |  |  |  |
| Discovery cohort = 1^st^ half |  | 28 of 30 | 17 (60.7%) | 93% | 94% / 91% | 0.0000057 |
| Validation cohort = 2^nd^ half |  | 27 of 29 | 17 (63.0%) | 67% | 77% / 50% | 0.0833 NS |
|  |  |  |  |  |  |  |
| Discovery cohort = 2^nd^ half |  | 27 of 29 | 17 (63.0%) | 89% | 88% / 90% | 0.0000531 |
| Validation cohort = 1^st^ half |  | 28 of 30 | 17 (60.7%) | 75% | 71% / 82% | 0.0082 |
|  |  |  |  |  |  |  |
| Cluster C  N=95 | Histories of CKD and diuretic use, age at asymptomatic diastolic dysfunction, indexed end systolic volume |  |  |  |  |  |
| Discovery cohort = 1^st^ half |  | 40 of 48 | 15 (37.5%) | 73% | 73% / 72% | 0.0044 |
| Validation cohort = 2^nd^ half |  | 35 of 47 | 16 (45.7%) | 74% | 63% / 84% | 0.0041 |
|  |  |  |  |  |  |  |
| Discovery cohort = 2^nd^ half |  | 35 of 47 | 16 (45.7%) | 77% | 75% / 79% | 0.0013 |
| Validation cohort = 1^st^ half |  | 40 of 48 | 15 (37.5%) | 68% | 73% / 64% | 0.0269 |
|  |  |  |  |  |  |  |
| Mixed |  |  |  |  |  |  |
| Discovery cohort = Entire Cluster B | Histories of diabetes, CKD, and diuretic use, AoV max gradient, diastolic wall strain | 55 of 59 | 34 (61.8%) | 76% | 76% / 76% | 0.0000922 |
| Validation cohort = Entire Cluster C |  | 83 of 95 | 39 (47.0%) | 64% | 77% / 52% | 0.0116 |
|  |  |  |  |  |  |  |
| Discovery cohort = Entire Cluster C | Histories of CKD and diuretic use, age at asymptomatic diastolic dysfunction, indexed end systolic volume | 75 of 95 | 31 (41.3%) | 77% | 77% / 77% | 0.0000022 |
| Validation cohort = Entire Cluster B |  | 47 of 59 | 28 (59.6%) | 55% | 43% / 74% | 0.4658 NS |

**ntire Cluster C
tmenablerogressiion of initial diagnosis of diastolic .te the feasibility of using machine learning to p
on cohorts were greater than a ns) Figure 2 have been modified d not be xxxxdata however any results from this cluster have beeSupplementary Table 2 legend:**

To test the predictive models each population (entire population, cluster B or cluster C) was divided into two cohorts, a discovery cohort and a validation cohort. Since sample size was small, variables for the cross validation study were assumed to be those found for the entire cohort. Predictive parameters for HFpEF outcomes for the discovery cohort were derived, and then the prediction was tested on the validation cohort.

For the entire population, logistic regression coefficients, using the variables previously found to be predictive of HFpEF outcome (histories of diabetes, CKD, atrial fibrillation, and diuretic use), were recalculated for the first half (discovery cohort). Using these coefficients, predictions of HFpEF outcome for each patient were calculated for both the discovery and validation cohorts. The number of correct predictions (progression to HFpEF plus remaining asymptomatic) as well as sensitivity and specificity of the prediction was determined. The same process was then repeated using the second half as the discovery cohort and the first half as the validation cohort.

A similar process was used for cluster B using variables (histories of diabetes, CKD and diuretic use, AoV max gradient, and diastolic wall strain), and for cluster C using variables (histories of CKD and diuretic use, age at asymptomatic diastolic dysfunction, and indexed end systolic volume).

Likewise parameters derived for cluster B were applied to cluster C and parameters for cluster C were applied to cluster B.

All predictions based on a discovery cohort and then applied to both the discovery and validation cohorts were greater than random predictions of 50% with two exceptions. Prediction based on the first half of Cluster B failed to accurately predict the second half, however predictions based on the second half did predict the outcomes of the first half. Predictions based on characteristics of Cluster C failed to accurately predict the outcomes of Cluster B. In general, the predictions for the discovery cohorts were higher than the predictions for the validation cohorts. The lowest predictions were found when optimum predictive parameters for cluster B were applied to cluster C and vice versa.

Within the same population (entire group, cluster B or cluster C) the discovery cohort can reasonably predict HFpEF outcome for the validation cohort. However, using cluster B population to predict cluster C population (or vice versa) yields poorer predictions.

* The weighting (coefficient) determined for the logistic regression depends on the frequency/value of the variable in the discovery cohort so the logistic regression is population-dependent.

** The cutoff for the logistic regression prediction is dependent on the frequency of the outcome in the discovery cohort and is population-dependent.

NS = not significant
